# Porcine Cross-Linked Collagen Matrix for Peri-Implant Vertical Soft Tissue Augmentation: A Randomized Prospective Observational Study

**DOI:** 10.3390/jfb15090261

**Published:** 2024-09-10

**Authors:** Giorgio Tabanella, Massimiliano Viale

**Affiliations:** 1 O.R.E.C.—Oral Reconstruction and Education Center, Via Rovereto 6, 00198 Rome, Italy; 2 Institute for Complex Systems—CNR, P.le Aldo Moro 2, 00185 Rome, Italy; massimiliano.viale@gmail.com

**Keywords:** peri-implant mucosa, emergence profile, soft tissue augmentation, vertical soft tissue augmentation, horizontal soft tissue augmentation, papilla regeneration, 3D soft tissue augmentation, periodontal plastic surgery, mucosa phenotype boosting, collagen matrix, papilla regeneration

## Abstract

The mucosa height has always been of interest in modern implant dentistry to obtain biomimetic results. Papilla height, mucosa scalloping, and free mucosal margin level are crucial to achieve “pink aesthetics”. The aim of this study was to investigate the vertical increase in the peri-implant soft tissues with a porcine cross-linked collagen matrix (Geistlich Fibro-Gide^®^). **Methods:** A total of 60 patients were divided into the following three groups: Group 1—patients who received porcine cross-linked collagen matrix for vertical soft tissue augmentation and a cover screw combined with a coronally advanced flap (CAF); Group 2—patients who received the collagen matrix combined with a healing abutment and CAF; Group 3 (control group)—patients who received a traditional surgical approach based on crestal incision and no collagen matrix as well as no CAF. **Results:** The average horizontal tissue thickness growth after 3 months was more effective for Group 1 (1.35 ± 1.23 mm) compared to Group 2 (0.85 ± 0.67 mm) and the control group (0.20 ± 0.41 mm). The average tissue height growth was 1.05 ± 1.39 mm for Group 1, 0.32 ± 1.28 mm for Group 2, and −0.05 ± 0.39 mm for the control group. Finally, the average increase in the band of keratinized mucosa was 0.60 ± 1.23 mm for Group 1, −0.60 ± 0.94 mm for Group 2, and 0.45 ± 0.60 mm for the control group. **Conclusions:** The combination of the CAF, porcine cross-linked collagen matrix, and cover screw resulted in better clinical results compared to Group 2 and 3.

## 1. Introduction

The peri-implant mucosa plays a critical role in the success and longevity of endosseous dental implants [1]. This specialized tissue interface between the implant and the surrounding oral environment not only facilitates osseointegration but also helps in maintaining the health and stability of the implant over time [2,3,4,5]. The condition of the peri-implant mucosa, including its thickness, quality, and vascularity significantly influences the success of dental implant treatment [4]. A thick and healthy peri-implant mucosa provides better support for the peri-implant soft tissues, minimizing the risk of complications such as bone loss, mucosal recession and implant exposure [5].

However, if it is true that the horizontal thickness of the peri-implant mucosa significantly affects the biomechanical behavior, biological response, and aesthetic outcome of endosseous dental implants, very little has been reported in the scientific literature about the significance of the vertical thickness as well as the mucosa height [3,6,7,8]. The vertical thickness of the mucosa is the measurement from the implant platform to the mucosa facing the oral cavity before the uncovery of the implant, whereas the mucosa height is the distance, after implant uncovery, from the implant platform to the free mucosal margin, which is the vertical dimension of the connective tissue, the junctional epithelium, and the sulcus above the implant platform. Both measurements are correlated to each other and very close in dimensions.

Accordingly, the vertical dimension of the peri-implant mucosa represents the transmucosal compartment directly in contact with the emergence profile [9] of the definitive restoration. This transitional zone between the implant crown and the surrounding soft tissues plays a crucial role to influence both the aesthetics and longevity of dental implants [9]. In fact, the emergence profile directly affects the visual appearance of the implant-supported restoration; it contributes to the stability and health of the peri-implant soft tissues, minimizing the risk of soft tissue recession, inflammation, and the loss of attachment and, finally, it significantly influences patient satisfaction and quality of life following implant treatment [9].

Several techniques have been proposed to horizontally augment the peri-implant mucosa [10,11,12,13,14] to create an ideal soft tissue environment for implant placement. However, few papers have reported techniques to increase the vertical thickness of the mucosa around dental implants [9,15]. In fact, whereas horizontal mucosa augmentation has been considered more relevant because of its capabilities to reduce peri-implant bone loss, vertical soft tissue augmentation has mainly been related to “pink aesthetics” and considered to be of less importance. Although Thoma et al. [9] introduced the application of biomaterials to increase the mucosa height and Verardi et al. [15] suggested the use of biomaterial in contact with the implant head, no other studies have compared the use of biomaterials in conjunction with coronally advanced flap and the use of cover screws or healing abutment.

The aim of this study was thus to investigate a novel approach to increase the vertical thickness of the peri-implant mucosa with a porcine cross-linked collagen matrix (Geistlich Fibro-Gide^®^) combined with a coronally advanced flap (CAF) and a cover screw or a healing abutment versus the traditional approach with no soft tissue grafting and no CAF management.

## 2. Materials and Methods

This randomized prospective clinical study included 60 patients who received single endosseous dental implants. Patients were divided into the following three different groups based on the type of surgical technique utilized at second stage surgery: Group 1—patients who received porcine cross-linked collagen matrix for vertical soft tissue augmentation and a cover screw combined with a coronally advanced flap; Group 2—patients who received collagen matrix combined with a healing abutment and CAF; and Group 3 (control group)—patients who received a traditional surgical approach based on crestal incision and no collagen matrix as well as no CAF. The healing abutment used in Group 2 was 3 mm in height in order to clearly show a clinical evident difference compared to Group 1 (collagen matrix combined with cover screw and CAF).

All clinical procedures were performed in full compliance with the Declaration of Helsinki, and the subsequent revisions (Fortaleza 2013) and study protocol were approved by the Ethical Committee Lazio 1 (Prot. N. 1396/CE Lazio 1).

All patients were consecutively enrolled and assigned to a group based on a randomization software (random.org). A random generator software assigned each patient to a specific group after having specified the total patient number of patients and the numbers of patients for each group.

Before enrolling the patients, they were asked to sign the informed consent and informed about the therapeutic alternatives.

The inclusion criteria were as follows: (1) need for an implant-supported single-crown restoration; (2) >21 years of age; (3) any type of definitive abutment; (4) single cemented or screw-retained definitive restorations; and (5) mono-edentulism. The exclusion criteria included the following: (1) any systemic disorder or medication known to alter bone metabolism; (2) infection of the implant site; (3) probing depth > 4 mm at natural teeth; (4) inadequate oral hygiene; (5) pregnancy: (6) lactation; and (7) uncontrolled medical conditions such as diabetes mellitus. Sample size calculation was based on comparable clinical studies [16].

At the second-stage surgery, an incision was executed in the middle of the crest and the following measurements were performed by one examiner (G.T.) at each implant site which was considered as a statistical unit: (1) the height of the keratinized tissue from the mucogingival junction to the free mucosal margin through a periodontal probe (Hu-Friedy^®^ Color-Coded Single-End Unc Probe 1-15, Chicago, IL, USA) on the medial aspect of the implant; (2) the thickness of the mucosa which was measured with a caliper (Hu-Friedy^®^ Castroviejo Caliper, Chicago, IL, USA), considering the medial aspect of implant platform as a point of reference; (3) the height of the mucosa which was measured with the same periodontal probe from the medial aspect of the implant platform to the free mucosal margin. Each implant was previously inserted at the same level of the crestal bone in order to avoid false measurements related to the depth of insertion. Probe measurements were rounded off to the nearest millimeter. Before each second-stage surgery, the site was scanned using an intraoral scanner (iTero Elements Plus Series^®^, Align Technology, Inc., San Jose, CA, USA)) in order to obtain multiple stl files to analyze the mucosa 3D augmentation at 1, 3, and 12 months after the periodontal plastic surgery. The overlapping of the stl files allowed us to analyze the maturation, migration, thickening, or recession of the soft tissue over a period of 12 months.

### 2.1. Surgical Techniques

**Figure 1:**
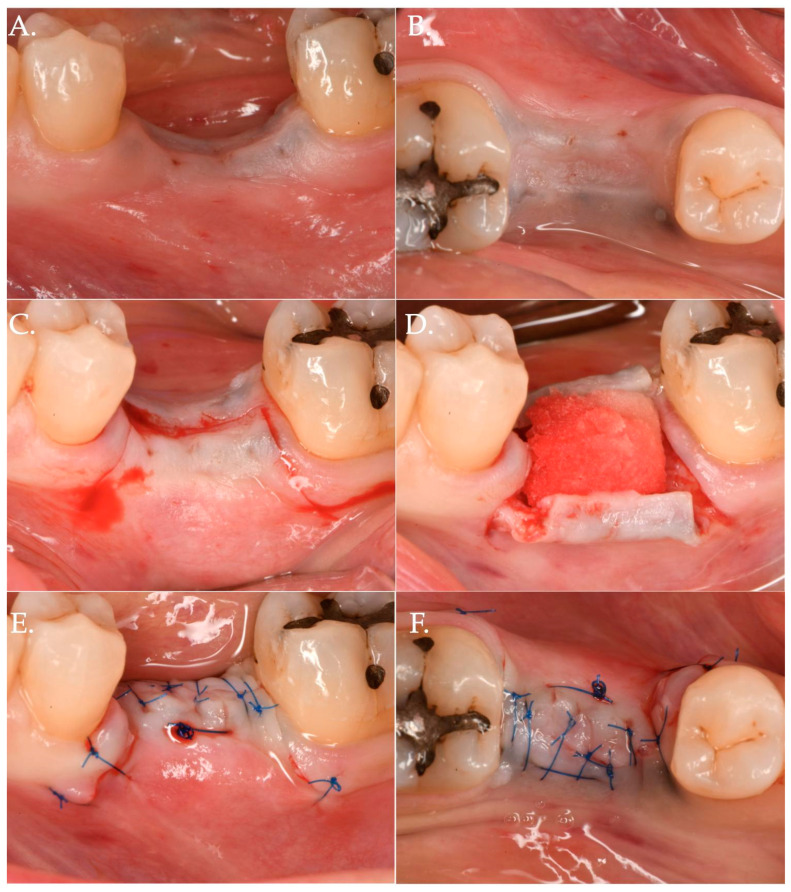
Clinical case related to 1 of the 20 patients of Group 1 patients who received Geistlich Fibro-Gide^®^ combined with a cover screw and CAF. The pre-op (Frame **A**,**B**) clinical view shows a vertical and horizontal deficiency of the soft tissue. A crestal incision, combined with two vertical releasing incisions (Frame **C**), was executed to access the implant platform. (Frame **D**) shows the collagen matrix of 5 mm of thickness positioned crestally. Micro 6-0 polypropylene sutures (Frame **E**,**F**) were executed to precisely close the wound allowing a primary intention healing of the coronally positioned flap.

**Figure 2:**
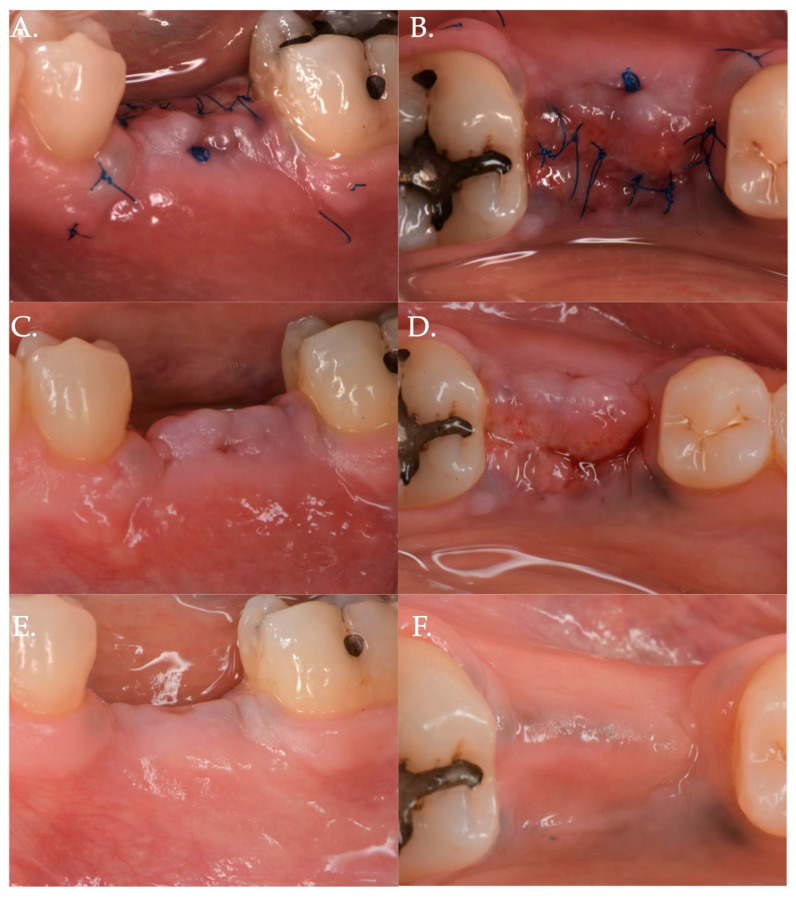
Post-operative healing after Geistlich Fibro-Gide^®^ combined with cover screw and CAF: 1 week (Frame **A**,**B**), 2 weeks (Frame **C**,**D**), and 3 months post-op (Frame **E**,**F**).

**Figure 3:**
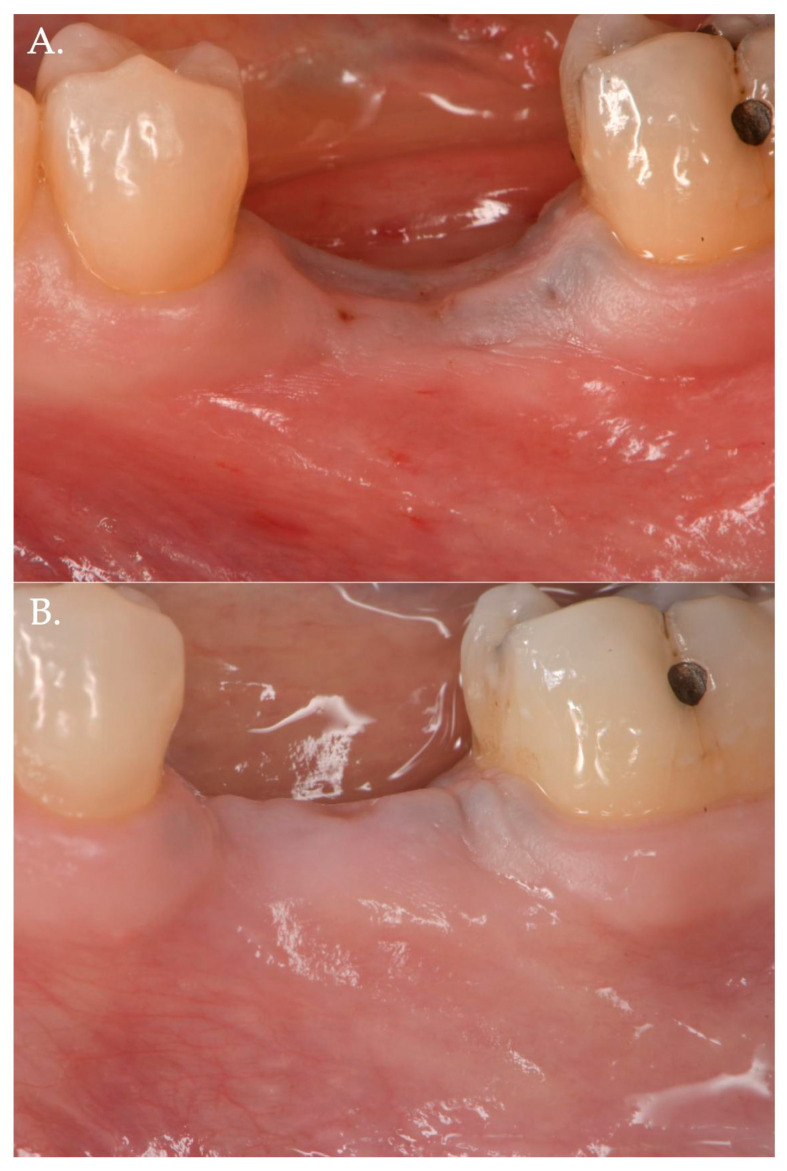
Before (Frame **A**) and after (Frame **B**) Geistlich Fibro-Gide^®^ combined with a cover screw and CAF. The post-operative clinical picture shows a significant improvement in the “pink aesthetics” in the vertical dimension.

**Figure 4:**
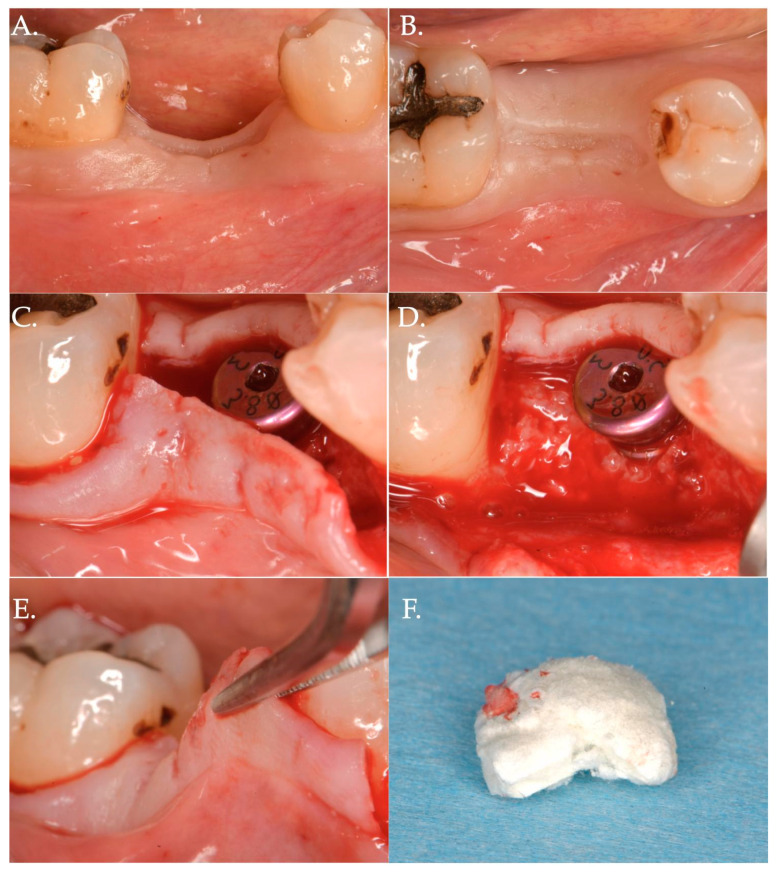
Surgical steps of a patient of Group 2—porcine cross-linked collagen matrix for vertical soft tissue augmentation and a healing abutment combined with a coronally advanced flap (CAF). The pre-surgical pictures demonstrate vertical and horizontal deficiency of the mucosa (Frame **A**,**B**). A full thickness flap is raised to get access to the implant platform and position a healing abutment of 3 mm height (Frame **C**,**D**). The flap is then released to obtain the necessary advancement (Frame **E**) and a collagen matrix of 5 mm in thickness is positioned above the healing abutment (Frame **F**).

**Figure 5:**
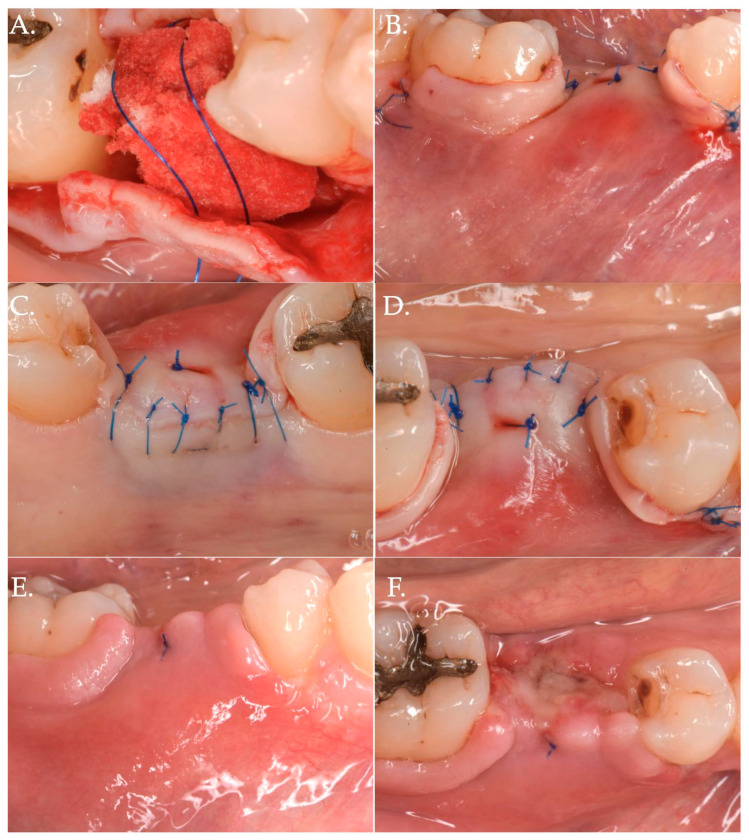
The collagen matrix is positioned coronally to the healing abutment and secured with 6.0 polypropylene sutures (Frame **A**). The CAF is secured coronally (Frame **B**) and microsutures (Frame **C**,**D**) maintain in position the flap. The clinical aspect after 1 week (Frame **E**,**F**) is demonstrating a delayed healing. This delay was very likely due to the excessive stretching of the advanced flap of more than 8 mm (5 mm matrix thickness + 3 mm healing abutment) compared to the bony crest.

**Figure 6:**
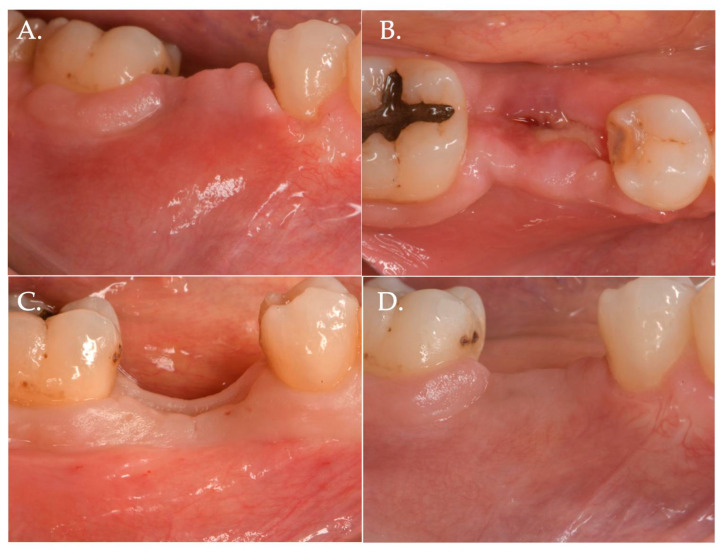
Healing after 2 weeks (Frame **A**,**B**) and 3 months (Frame **C**,**D**): The result is a significant improvement in the vertical “pink aesthetics”.

In Group 1, a crestal incision was executed in combination with two vertical releasing incisions in order to properly advance the flap in a coronal position (Figure 1, Figure 2 and Figure 3). The collagen matrix was then positioned on the cover screw and microsutures were performed to secure the flap in a coronal position as well as allowing a first intention healing of the mucosa. The collagen matrix was not fixated with pins, screws, or periosteal sutures since the pressure related to the microsuturing was enough to avoid a shifting of the matrix. In Group 2, the same surgical approach was used, except for the fact that a healing abutment was screwed onto the implant platform (Figure 4, Figure 5 and Figure 6). In the control group (Group 3), only a crestal incision was performed to get access to the implant platform without CAF and collagen matrix.

### 2.2. Stl Files Measurements

**Figure 7:**
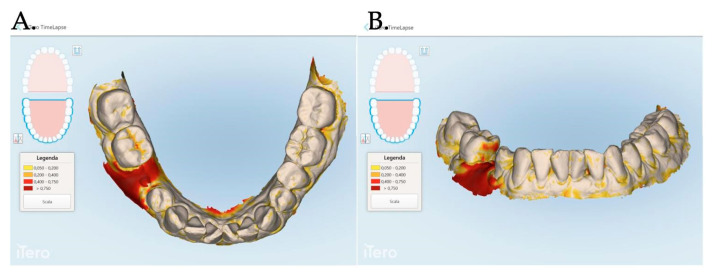

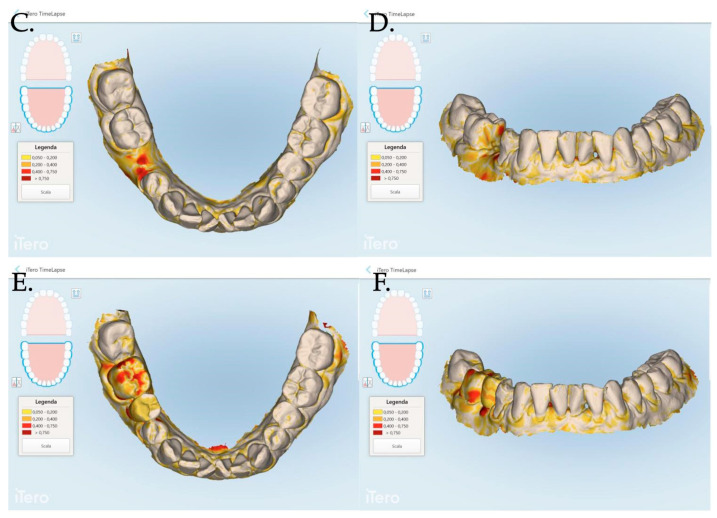
The Stl file overlapping and digital soft tissue mapping allowed us to monitor and measure the volume changes of the achieved results after 1 month (**A**,**B**), between 0 and 3 months (**C**,**D**), between and 0 and 12 months (**E**,**F**). A patient of Group 1 is reported in the digital reconstructions.

**Figure 8:**
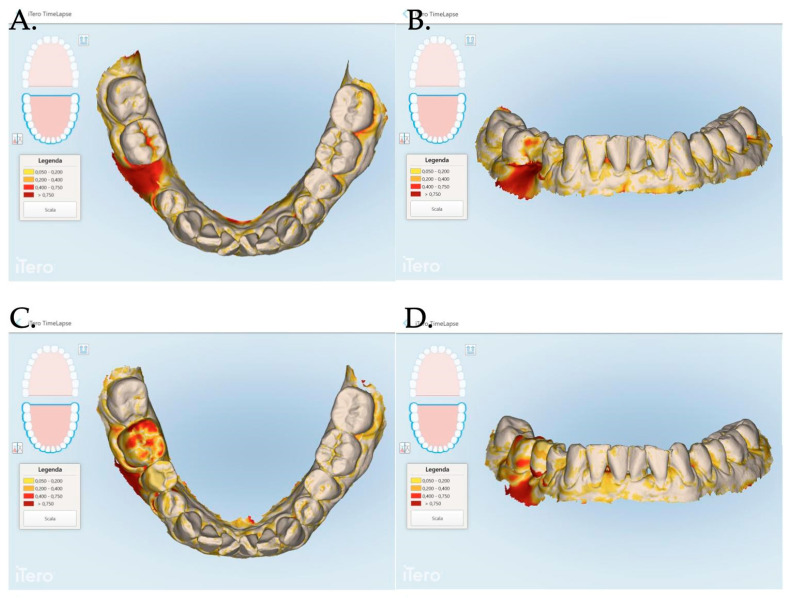

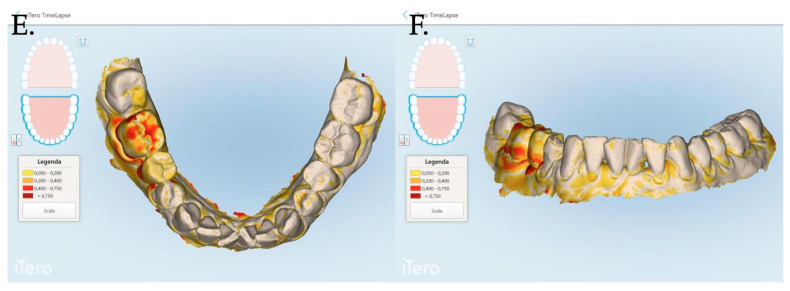
Stl files analyses between 1 and 3 months post muco-gingival plastic surgery (**A**,**B**), 1 and 12 months (**C**,**D**) and 3 and 12 months (**E**,**F**). The same patient of Figure 7 is reported in the above digital reconstruction.

An intraoral scanner (iTero Elements Plus Series^®^) was used to scan each patient at each time point: 0, 1, 3, and 12 months after mucogingival plastic surgery. The iTero software (Plus Computing Unit 2.8.15.70 ) was able to precisely measure the increase or decrease in the mucosa volume by overlapping the stl files. The software was also able to provide a difference in color to better visualize the 3D mucosa changes (Figure 7 and Figure 8).

## 3. Statistical Analysis—Spearman’s Rank

For the same patients, the effect of the clinical evolution was evaluated by looking at an index that summarized the progress of mucosal strengthening one year after the mucogingival plastic surgery—Spearman’s rank correlation index. The Stl files were produced before soft tissue augmentation and at 1, 3, and 12 months after second-stage surgery to measure the horizontal and vertical regeneration of the mucosa.

In order to evaluate the mucosa volume augmentation, Spearman’s rank correlation coefficient of the pair series X_i_, Y_i_ was used. Spearman’s index is worth +1 if there is a correlation (grows or have the same value with increasing) and −1 if there is an anti-correlation (decreases with increasing) regardless of if it is linear or not. Spearman’s index of y = e^x^ sampling (certainly a very increasing function for x > 0) is always 1.

## 4. Results

A total of 60 (28 females and 32 males) patients were included in the study. Patients ranged between 22 and 72 years of age and 10 patients were smokers.

For each patient, the area of interest was observed by overlapping the stl files at the time of surgery and at 1, 3, and 12 months after the mucosa management in order to analyze the augmentation as well as the creeping (vertical mucosa movement), thickening, and stabilization of the obtained results. The stl files overlapping allowed us to evaluate the 3D soft tissue augmentation that includes the simultaneous horizontal and vertical augmentation.

**Figure 9:**
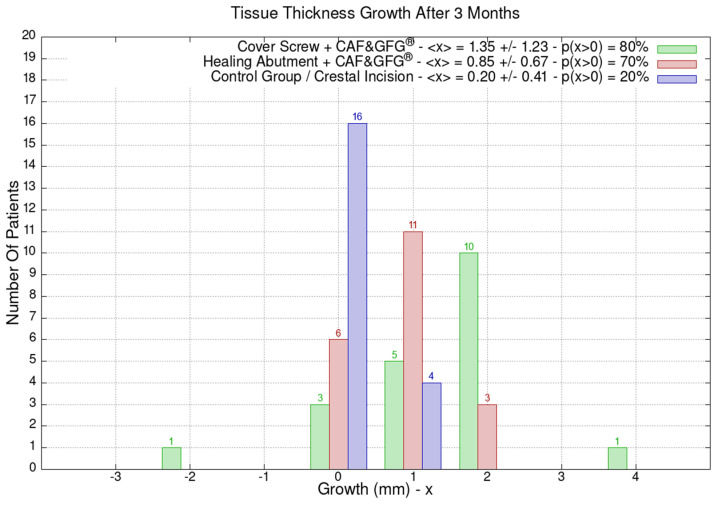

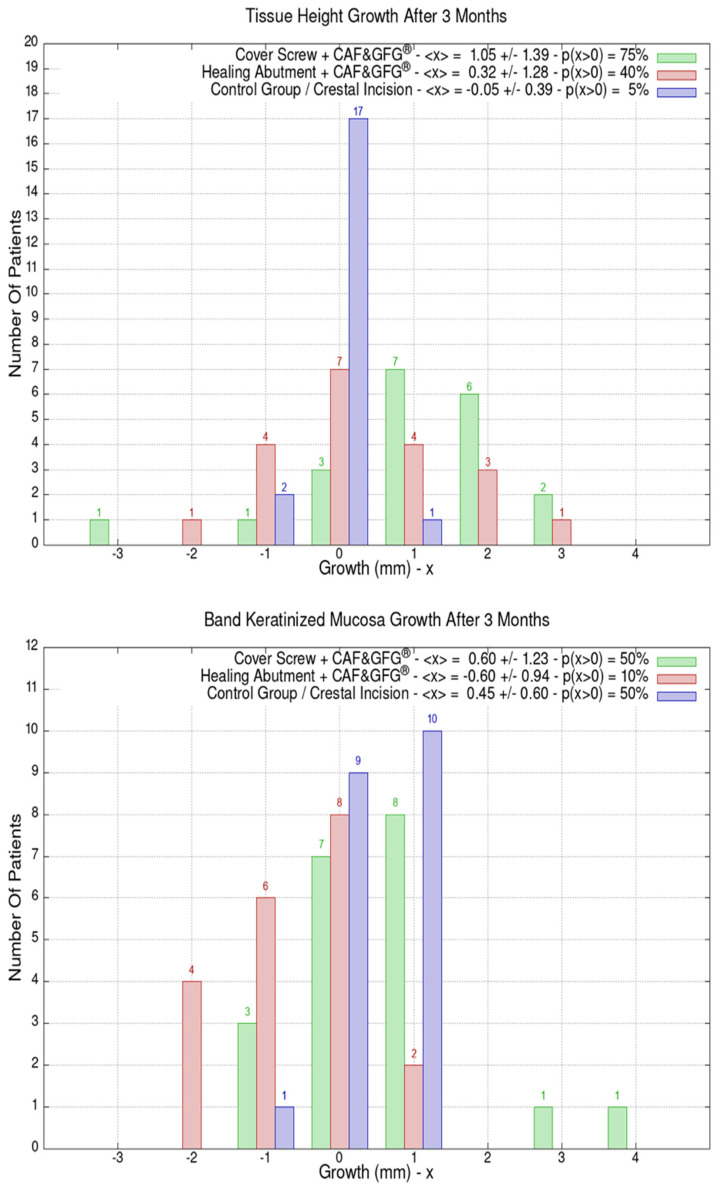
Mucosa thickness, height, and keratinized tissue changes 3 months after mucogingival plastic surgery. Divided by groups, the number of patients is shown as a function of the millimeters of growth or decrease. In the plot keys, the average growth value <x> and the percentage of patients p(x > 0) who have had a strictly positive evolution are reported. It is evident that the group treated with the “Cover Screw Implant combined with CAF and collagen matrix” (green) had a better evolution compared to both the one treated with the “Healing Abutment Implant combined with CAF and collagen matrix” (red) and the control one (blue) for which we performed a crestal incision only.

The height and the thickness of the mucosa as well as the band of keratinized tissue were measured at the time of surgery and after 3 months in order to evaluate its direct growth correlated to the surgical technique which was used to increase the phenotype’s thickness and volume. The 60 patients were divided into the following three groups of 20 patients each based on the type of surgery they underwent: green (Group 1) for the cover screw combined with CAF and collagen matrix, red (Group 2) for healing abutment combined with CAF and collagen matrix, blue (Group 3) for the control group for which we performed a crestal incision. Figure 9 is clearly showing that the group treated with “Cover Screw Implant combined with CAF and collagen matrix” (green) had a more favorable evolution compared to the other two groups such as the one treated with “Healing Abutment Implant combined with CAF and collagen matrix” (red) and the control group (blue).

The average horizontal tissue thickness growth after 3 months was more effective for Group 1 (1.35 ± 1.23 mm) compared to Group 2 (0.85 ± 0.67 mm) and the control group (0.20 ± 0.41 mm). The average tissue height growth was 1.05 ± 1.39 mm for Group 1, 0.32 ± 1.28 mm for Group 2, and −0.05 ± 0.39 mm for the control group. Finally, the average increase in the band of keratinized mucosa was 0.60 ± 1.23 mm for Group 1, −0.60 ± 0.94 mm for Group 2, and 0.45 ± 0.60 mm for the control group. Group 1 resulted in better clinical results compared to Group 2 and 3 (Figure 9).

**Figure 10:**
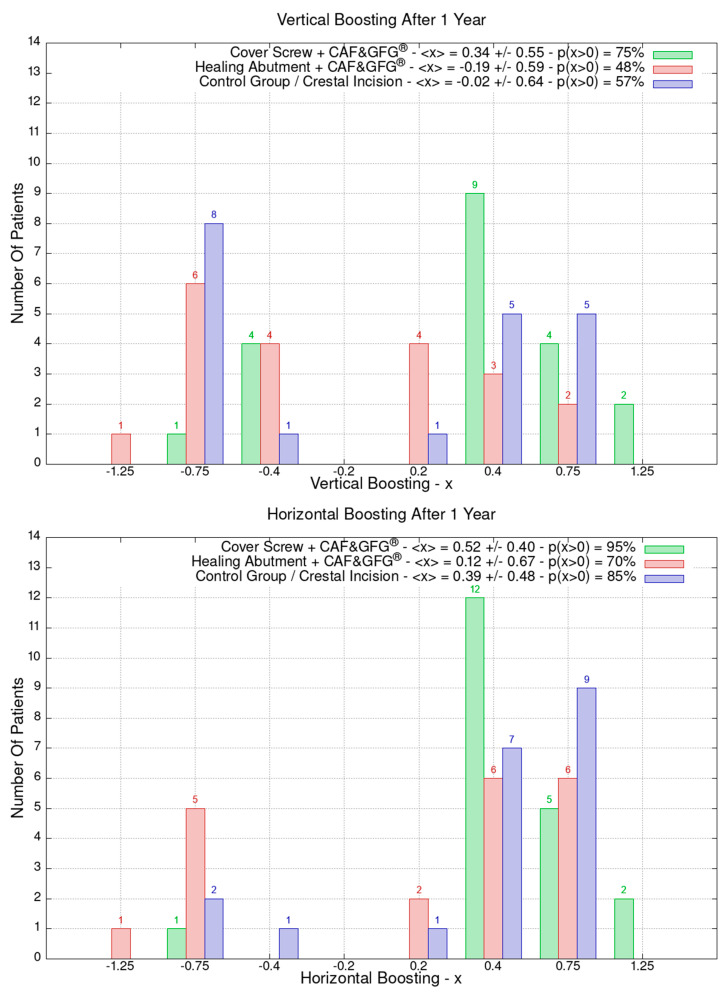
Vertical and horizontal boosting 1 year after surgery. As for Figure 1, divided by groups, the number of patients is reported as a function of boosting. In the plot keys, the average boosting <x> and the percentage of patients p(x > 0) who have had a strictly positive boosting are presented.

**Figure 11:**
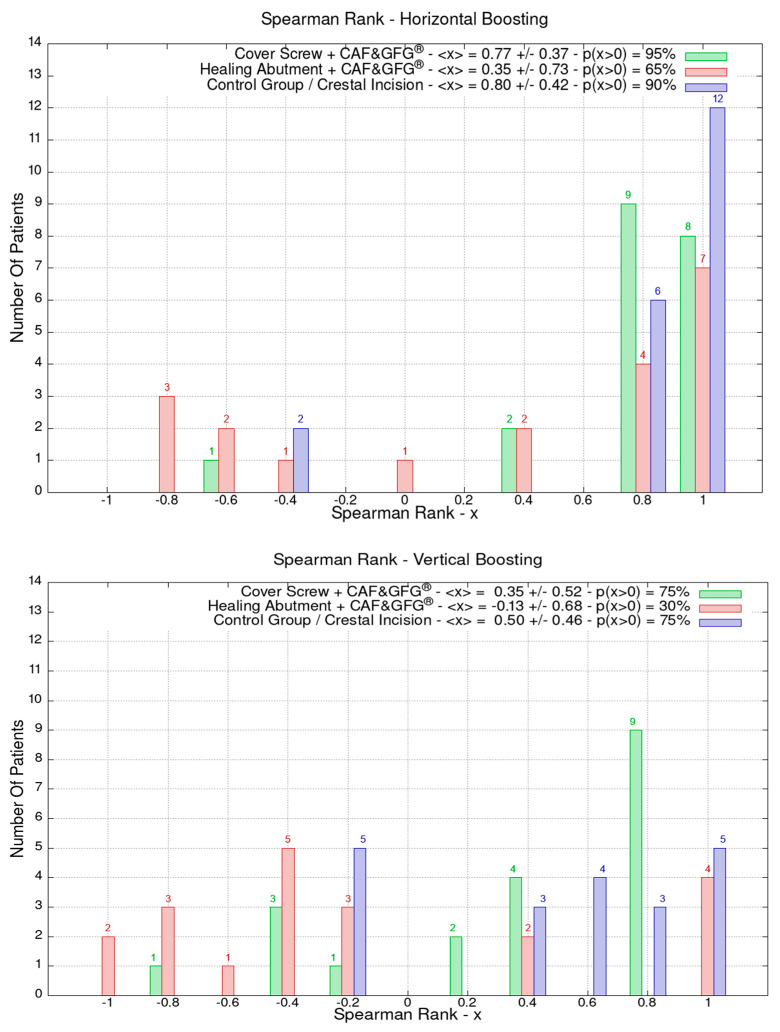
The vertical and horizontal augmentation is described using Spearman’s rank. The values close to 1 represent the highest performing results. The vertical and soft tissue augmentation is significantly higher when the cover screw was used and when a coronally positioned flap was combined with collagen matrix.

The data related to the Stl file analysis are reported in Figure 10. The continuous boosting of and improvement in the mucosa are also evident in this data analysis (Figure 11). In particular, the greater effectiveness of the “Cover Screw Implant combined with CAF and collagen matrix” is concrete but less prominent than the tissue growth shown in Figure 9. Interestingly, after 1 year, the control group (blue) had a slightly better evolution than the one treated with the “Healing Abutment Implant combined with CAF and collagen matrix” (red).

## 5. Discussion

The emergence of dental implants as a reliable treatment modality for replacing missing teeth has revolutionized the field of implant dentistry. However, achieving optimal aesthetic outcomes with dental implants, particularly in the anterior maxilla, remains a challenge due to the complex interplay of hard and soft tissue dynamics. Soft tissue augmentation techniques have emerged as valuable tools for enhancing peri-implant soft tissue volume and contour, thereby improving the aesthetic outcomes and long-term implant stability. This stability is crucial for maintaining implant health and preventing complications such as peri-implant mucositis and peri-implantitis, which can compromise the longevity of the implant. The “pink aesthetics” must match with the “white aesthetics” so that the patients’ aesthetic outcomes and natural-looking restorations blend with their existing dentition. Furthermore, a well-designed emergence profile contributes to a confident smile, improved self-esteem, and overall satisfaction with the implant-supported restoration. Scientifically, the thickness of the peri-implant mucosa influences the biomechanical and biological responses at the implant interface, impacting osseointegration and long-term stability. In particular, the importance of the peri-implant mucosa is related to the following:

- Biomechanical Stability: The thickness of the peri-implant mucosa provides mechanical support to the soft tissue, distributing occlusal forces evenly and reducing stress concentration around the implant–abutment complex [17]. Thicker mucosa can better resist mechanical trauma and prevent soft tissue recession, thereby preserving the integrity of the peri-implant soft tissues [18,19].
- Biological Response: Thicker peri-implant mucosa enhances the blood supply and vascularity, promoting optimal wound healing and tissue adaptation to the implant surface. Adequate vascularization facilitates nutrient and oxygen delivery to the surrounding tissues, fostering cellular proliferation and collagen deposition, which are essential for osseointegration and soft tissue integration [17,20,21].
- Aesthetic Outcome: The thickness of the peri-implant mucosa directly influences the [22] stability and contour of the peri-implant soft tissues, which are critical for achieving optimal aesthetic outcomes. Thicker mucosa provides better support for the papillae and gingival architecture, minimizing the risk of mucosal recession and ensuring harmonious gingival contours around the implant restoration [4,5,23,24].
- Implant Emergence Profile: The soft tissue thickness influences the emergence profile of the implant restoration, which determines the transition zone between the implant crown and the surrounding soft tissues. A thicker mucosa allows for better emergence profile management, resulting in a more natural-looking and harmonious integration of the restoration with the gingival architecture [25,26]. Similarly, the implant crown volume and its angulation of no more than 30° compared to the long axis of the implant may be responsible for an excessive compression of the achieved mucosa volume. Both parameters, mucosa and crown volume, may influence the final aesthetic and functional outcome.

Although, the vertical soft tissue thickness of the mucosa around endosseous dental implants may play a critical role in ensuring implant success, stability, and aesthetics, the scientific literature underscores the significance of vertical soft tissue thickness in various aspects of implant dentistry, including biomechanics, biological response, and aesthetic outcome. In our study, we reported two different approaches to vertically augment the peri-implant mucosa. The use of cross-linked collagen matrices is becoming more popular due to less invasive surgical procedures and results which are comparable with those achieved with connective tissue grafts [22,27,28,29]. The results of our study clearly demonstrated a substantial positive evolution, on average and in percentage, for patients treated with the cover screw combined with CAF and collagen matrix and a negative evolution, also compared to the control group, for patients treated with the healing abutment combined with the CAF and collagen matrix. The low effectiveness of the healing abutment combined with the CAF and collagen matrix is evident, but this could be related to the excessive stretching of the mucosa to completely cover the standard concave healing abutment of 3 mm. If it is considered that the thickness of the collagen matrix utilized in this study was 5 mm, a CAF of more than 8 mm would have been executed to completely cover the healing abutment. This procedure could have led to an excessive coronal repositioning of the flap and an evident low blood supply. In order to properly study the mucosa, all implants were placed crestally so that the depth of insertion could not influence the mucosa height and the biologic width reformation. We also reported that the soft tissue augmentation that was achieved was vertical and horizontal thus allowing us to obtain a simultaneous good quality and quantity of the peri-implant mucosa to face the emergence profile as well as the oral cavity. Compared to the other surgical techniques [30,31,32] that have been proposed to improve the peri-implant mucosa, our approach was minimally invasive and asymptomatic thus allowing us to achieve not only horizontal thickness but also an increase in the band of keratinized mucosa as well as a higher mucosa dimension facing the definitive restoration. Furthermore, the stl file analyses demonstrated the continuous volume increase, creeping (soft tissue growth in areas where inaccessible spaces were either intentionally created or naturally present), and stability of the achieved mucosa volume for the follow-up period of 12 months.

In summary, excessive stress of the mucosa during a coronally repositioned flap should be avoided but, at the same time, it is not advisable to still perform the traditional approach based on crestal incision at the time of uncovery since this could lead to a poor quality and quantity of the mucosa.

This study also showed how this simplified technique could be of help to switch from a thin to a thick phenotype thus potentially reducing the risk of complication in implant dentistry. However, the potential limitations of the study may be related to the reduced number of statistical units, the short period of follow-up, and the surgical technique which may be operator dependent. Furthermore, all measurements were taken by only one examiner (G.T.) thus being a limitation of this study. However, all measurements were executed as described in previous publications [6,11]. At the same time, we believe the strength of the study is the statistical analysis performed by the second author (M.V.). In fact, the blinding of the statistician in this study is an established approach to minimize the risk of performance and detection bias.

Finally, it is the authors’ opinion that digital technology should be used to monitor implant health over a long period of time. Digital soft tissue mapping had already been proposed in the previous publications [22,33]; however, the increasing importance of proper communication with patients, along with the potential legal issues, as well as the need to share information with other colleagues will definitely increase the use of this technology. In fact, digital soft tissue monitoring would allow for easy access to recorded data and shared information. The proposed protocol to monitor implant health would be minimally invasive compared to peri-apical radiographs and probing. Furthermore, the first approach would monitor only the peri-implant bone level. Similarly, the periodontal probing would disrupt the delicate adhesion of the peri-implant mucosa onto the abutment interface. On the patient side, it takes the communication to a visual level, allowing patients to see the differences which are often difficult to understand when talking about changes of a few millimeters.

Clinicians should carefully consider augmenting the peri-implant mucosa with atraumatic plastic surgery. The results of this study clearly demonstrated the excellent outcome to improve the quality and quantity of the vertical mucosa thickness when a coronally advanced flap was used in conjunction with a collagen matrix and a cover screw. The vertical soft tissue thickness of the mucosa around endosseous dental implants is a crucial determinant of implant success, stability, and aesthetics. Clinicians should consider this parameter during treatment planning and prosthetic design to optimize outcomes and patient satisfaction. Advancements in biomaterials, surgical techniques, and regenerative therapies will continue to expand the armamentarium of vertical soft tissue augmentation in implant dentistry. Future research efforts should focus on optimizing treatment protocols, exploring innovative biomaterials, and investigating the role of tissue engineering approaches in enhancing peri-implant soft tissue volume and contour. The results of this study show how new biomaterials can be of importance in soft tissue regeneration when combined with the proper surgical approach. In particular, the technique we described clearly allows us to improve the quality of the mucosa with a minimally invasive and biomimetic approach, simultaneously resulting in low morbidity with the implant placement or at second-stage surgery. The direct clinical application may be the immediate and simplified 3D soft tissue augmentation as well as the reduction in surgical interventions. Finally, the vertical positioning of the mucosa which has always been critical and difficult to reach may be significantly improved when the collagen matrix is used in combination with a cover screw and a coronally positioned flap. Ultimately, this approach may add an extra tool to significantly improve biomimetic results in the anterior maxilla such as papilla height, mucosa scalloping, and free mucosal margin levels.

## 6. Conclusions

The results of this study support the use of mucogingival plastic surgery to increase the volume as well as improve the quality of the peri-implant mucosa. In particular, among the different surgical techniques that have been proposed, the coronally advanced flap combined with porcine cross-linked collagen matrix and a cover screw resulted in more thickness, vertical soft tissue height, and an adequate band of keratinized tissue.

## Data Availability

The original contributions presented in the study are included in the article, further inquiries can be directed to the corresponding author.
